# Deacetylation of TFEB promotes fibrillar Aβ degradation by upregulating lysosomal biogenesis in microglia

**DOI:** 10.1007/s13238-016-0269-2

**Published:** 2016-05-21

**Authors:** Jintao Bao, Liangjun Zheng, Qi Zhang, Xinya Li, Xuefei Zhang, Zeyang Li, Xue Bai, Zhong Zhang, Wei Huo, Xuyang Zhao, Shujiang Shang, Qingsong Wang, Chen Zhang, Jianguo Ji

**Affiliations:** State Key Laboratory of Protein and Plant Gene Research, College of Life Sciences, Peking University, Beijing, 100871 China; Institute of Systems Biomedicine, Department of Pathology, School of Basic Medical Sciences, Center for Age-Related Diseases, Peking University Health Science Center, Beijing, 100191 China; State Key Laboratory of Biomembrane and Membrane Biotechnology, Peking University, Beijing, 100871 China

**Keywords:** Alzheimer’s disease, microglia, lysosomes, TFEB, SIRT1, deacetylation

## Abstract

Microglia play a pivotal role in clearance of Aβ by degrading them in lysosomes, countering amyloid plaque pathogenesis in Alzheimer’s disease (AD). Recent evidence suggests that lysosomal dysfunction leads to insufficient elimination of toxic protein aggregates. We tested whether enhancing lysosomal function with transcription factor EB (TFEB), an essential regulator modulating lysosomal pathways, would promote Aβ clearance in microglia. Here we show that microglial expression of TFEB facilitates fibrillar Aβ (fAβ) degradation and reduces deposited amyloid plaques, which are further enhanced by deacetylation of TFEB. Using mass spectrometry analysis, we firstly confirmed acetylation as a previously unreported modification of TFEB and found that SIRT1 directly interacted with and deacetylated TFEB at lysine residue 116. Subsequently, SIRT1 overexpression enhanced lysosomal function and fAβ degradation by upregulating transcriptional levels of TFEB downstream targets, which could be inhibited when TFEB was knocked down. Furthermore, overexpression of deacetylated TFEB at K116R mutant in microglia accelerated intracellular fAβ degradation by stimulating lysosomal biogenesis and greatly reduced the deposited amyloid plaques in the brain slices of APP/PS1 transgenic mice. Our findings reveal that deacetylation of TFEB could regulate lysosomal biogenesis and fAβ degradation, making microglial activation of TFEB a possible strategy for attenuating amyloid plaque deposition in AD.

**Electronic supplementary material:**

The online version of this article (doi:10.1007/s13238-016-0269-2) contains supplementary material, which is available to authorized users.

## Introduction

The most common form of Alzheimer’s disease (AD) appears sporadically during the aging process and is characterized by deposition of β-amyloid (Aβ) peptides within the brain. An imbalance between Aβ clearance and production results in its accumulation as amyloid plaques that usually lead to perturbations of neural network (Palop and Mucke, 2010; Querfurth and LaFerla, 2010). Recent studies indicated that the inefficient Aβ removal might be the significant underlying mechanism in sporadic AD (Mawuenyega et al., 2010). Therefore, how to stimulate Aβ degradation within the brain needs to be thoroughly investigated.

Microglia, the resident macrophages in the central nervous system (CNS), play a key role in surveying the brain for abnormalities and are quickly activated in response to certain stimuli such as cellular debris or misfolded proteins (Lucin and Wyss-Coray, 2009; Nimmerjahn et al., 2005). Previous studies indicated that microglia were recruited to and activated by amyloid plaque mainly containing fibrillar Aβ, which is the clinical hallmark of AD in advanced stage (Meyer-Luehmann et al., 2008; Rogers et al., 2002). Moreover, inhibition of microglia accumulation accelerates the burden of plaques deposition within the brain (El Khoury et al., 2007). Several lines of evidence showed that microglia take up and degrade both soluble Aβ (sAβ) and fibrillar Aβ (fAβ), which contributes to the clearance of Aβ in addition to extracellular proteolysis pathway (Lucin and Wyss-Coray, 2009). Recent studies revealed that lipidated apoliprotein E (ApoE) contributes to microglial degradation of sAβ through neprilysin (NEP), a process that is regulated by PPARγ/LXRs signalling pathway (Jiang et al., 2008; Mandrekar-Colucci et al., 2012). Despite these findings, little is known about the mechanisms underlying fAβ degradation within microglia.

Lysosomes are regarded as the critical cellular digestive compartments that are responsible for degradation and recycling of a variety of intracellular and extracellular metabolites, depending on their various soluble acidic hydrolases. Piles of work on this cellular organelle demonstrated that numerous lysosomal storage disorders were triggered by the inheritated deficits of specific lysosomal function (Settembre et al., 2013b). Apart from these early-onset genetic diseases, it is notable that lysosomal dysfunction always leads to various age-related neurodegenerative diseases such as Alzheimer’s (AD), Parkinson’s (PD) and Huntington’s diseases (HD) (Schultz et al., 2011). Therefore, upregulation of lysosomal biogenesis, especially in microglia, may exert beneficial effects on attenuating Aβ pathogenesis in AD.

Transcription factor EB (TFEB) acts as a master regulator of lysosomal function by inducing over 500 target genes related with lysosomal biogenesis and autophagy, which is induced by its translocation from cytoplasm to nucleus under certain cellular stresses (Sardiello et al., 2009; Settembre et al., 2011). Further studies revealed that activation of TFEB meets the cellular needs to protect against the toxicities of the abnormal aggregates. Researchers discovered that the neural toxicity from α-synuclein or htt-aggregates could be markedly ameliorated when TFEB is overexpressed or pharmacologically activated in PD or HD animal models, respectively (Decressac et al., 2013; Tsunemi et al., 2012). Recently, TFEB is suggested to be participated in the Aβ-induced pathogenesis of AD by regulating lysosomal pathway (Zhang and Zhao, 2015). Further evidence indicated that TFEB enhances lysosomal biogenesis in both astrocytes and neuron, contributing to increased sAβ clearance and reduced Aβ generation, respectively (Xiao et al., 2014; Xiao et al., 2015). Despite these influential observations, whether TFEB would function in microglia and the mechanisms underlying TFEB functional pathway, especially its upstream regulation within nucleus, remain to be elucidated.

Accumulating evidence suggests that protein acetylation plays a role in regulating lysosome-mediated autophagy process (Banreti et al., 2013). SIRT1 is the most conserved member of sirtuins, a family of NAD^+^-dependent protein deacetylases, and contributes to deacetylation of multiple mammalian transcription factors such as p53, FOXOs, and NF-κB in nucleus, making it a key metabolic regulator involved in a vast number of cellular processes associated with age-related diseases including AD (Guarente, 2011; Herskovits and Guarente, 2014). Previous work indicated that SIRT1 protects against Aβ toxicity and cognitive deficits in animal models mainly through inhibiting inflammatory responses in microglia, because overexpression of SIRT1 in microglia suppresses NF-κB-signaling while the reduced level of SIRT1 during the aging process of microglia, upregulates IL-1β expression (Chen et al., 2005; Cho et al., 2015). Apart from the inflammatory perspective, the molecular mechanisms underlying the protective effects of SIRT1 in microglia are still being investigated.

Considering the critical role of TFEB in lysosomal biogenesis, we intended to explore whether TFEB would increase microglial clearance of Aβ and unveil the mechanisms underlying the upstream regulation of TFEB in lysosomal biogenesis in microglia.

In this study, we firstly identify protein acetylation as a novel post-translational modification of TFEB and demonstrate that TFEB is deacetylated by SIRT1 at K116. Moreover, SIRT1-mediated deacetylation of TFEB facilitates microglial degradation of fAβ by enhancing lysosomal biogenesis. Therefore, we identify the upstream molecular mechanism by which deacetylation stimulates TFEB induction of lysosomal biogenesis to enhance microglial degradation of fAβ and reduction of amyloid plaques, suggesting a novel strategy for treatment of AD.

## Results

### TFEB enhances microglial degradation of fibrillar Aβ in lysosomes

**Figure 1:**
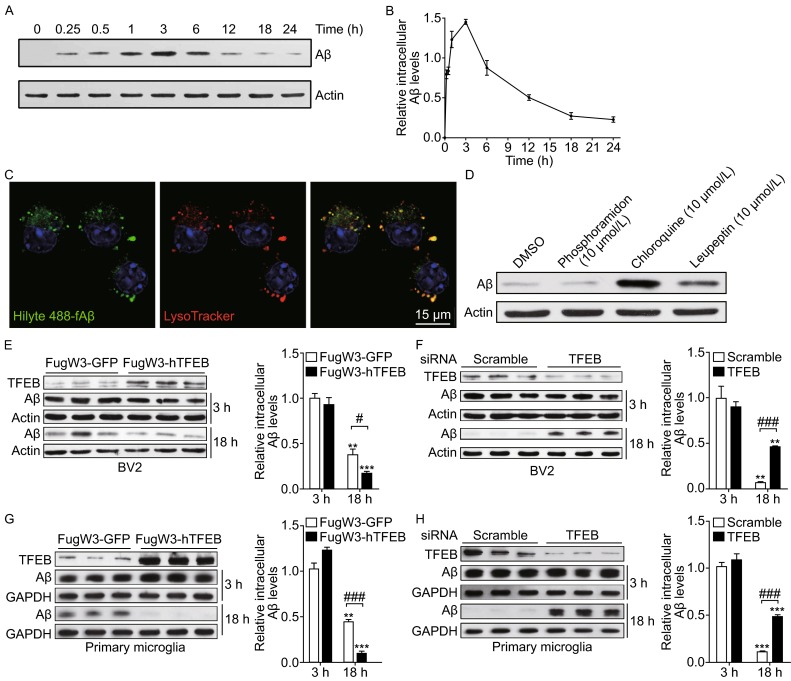
TFEB enhances microglial degradation of fibrillar Aβ in lysosomes. (A and B) Microglia internalize and efficiently degrade fibrillar Aβ. BV2 cells were incubated with fAβ (500 nmol/L) at 37°C and the cells were harvested and lysed at different time points, followed by detection of intracellular Aβ levels by Western blotting analysis (A). The band intensity was measured in three independent experiments indicating relative intracellular Aβ levels and the mean ± SEM are shown in (B). (C) Fibrillar Aβ is rapidly trafficked into lysosomes. Confocal imaging of live BV2 cells 30 min after addition of Hilyte488-labeled fAβ (500 nmol/L) showed localization of fAβ (Green) within lysosomes stained with LysoTracker (Red). Scale bar, 15 μm. (D) Internalized fAβ is degraded in lysosomes. Primary microglia from wild-type mice were pretreated with DMSO, Phosphoramidon (NEP inhibitor, 10 μmol/L), Chloroquine or Leupeptin (Lysosome inhibitor, 10 μmol/L) for 18 h. The cells were then incubated with fAβ (500 nmol/L) in the presence of DMSO or inhibitors for an additional 18 h. The band intensity was measured in three independent experiments indicating relative intracellular Aβ levels. (E and G) TFEB overexpression increases fAβ degradation in microglia. GFP or human TFEB was overexpressed in BV2 cells (E) or primary microglia (G) by lentiviral system. The cells were incubated with fAβ (500 nmol/L) for 3 h or 18 h and were harvested for detection of intracellular Aβ levels by Western blotting analysis. The band intensity was measured in three independent experiments indicating relative intracellular Aβ levels and the mean ± SEM are shown in the right panel. Two-way ANOVA, comparison between different time points, **P < 0.01; ***P < 0.001. Unpaired Student’s *t*-test, comparison against the Fugw3-GFP, #*P* < 0.05; ###*P* < 0.001. (F and H) TFEB knockdown inhibits fAβ degradation in microglia. Scramble or TFEB siRNA was delivered into BV2 cells (F) or primary microglia (H) for 72 h. The cells were treated as in (E and G). Two-way ANOVA, comparison between different time points, **P < 0.01; ***P < 0.001. Unpaired Student’s *t*-test, comparison against the Scramble, ###*P* < 0.001

**Figure 2:**
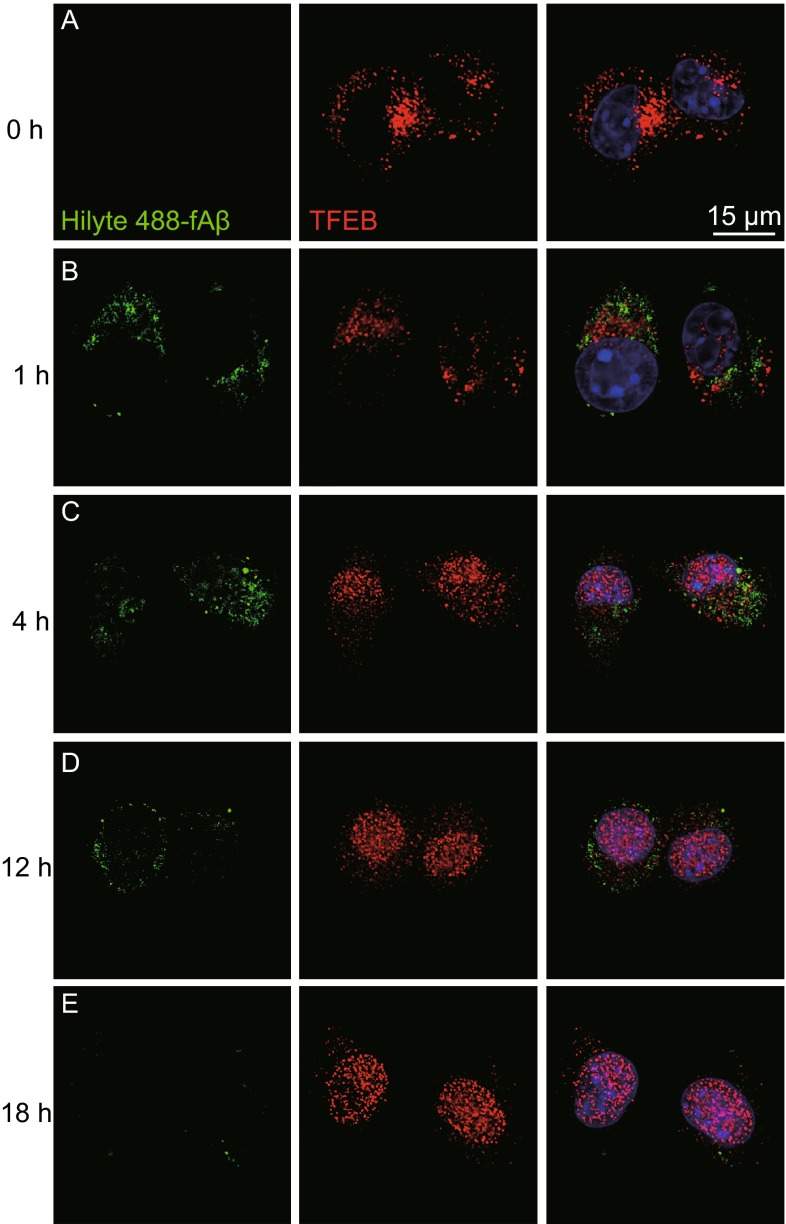
Fibrillar Aβ stimulates TFEB to translocate into nucleus. (A–E) TFEB translocates into nucleus under incubation of fibrillar Aβ. Confocal imaging of BV2 cells incubated with Hilyte488-labeled fAβ (500 nmol/L, Green) at different time points and immunostained for endogenous TFEB (Red). Scale bar, 15 μm

To investigate the contribution of microglia in clearance of fibrillar Aβ (fAβ), the major constituent of amyloid plaque deposited in AD brains, we first incubated fAβ that was generated and identified *in vitro* with BV2 cells and primary microglia (Ma et al., 2013). Our results revealed that fAβ was rapidly taken up and trafficked into lysosomes within 30 min (Fig. 1A–C). As time prolonged, the internalized fAβ level increased to the peak level at 3 h and then gradually disappeared at 18 h (Fig. 1B). By conducting this set of preliminary experiment, 3 h and 18 h were interpreted as the time points representing microglial capabilities of fAβ phagocytosis and degradation, respectively. Indeed, the fAβ originally added into the media was immediately and thoroughly internalized by microglia and little did we observe any resecretion in the media (Fig. S1A). Interestingly, we confirmed fAβ is exclusively degraded within lysosomes, for the reason that inhibitors of lysosomes such as chloroquine or leupeptin remarkably weaken microglial degradation of fAβ while phosphoramidon, inhibitor of NEP that is reported for sAβ degradation (Jiang et al., 2008), exerts little impact on this process (Fig. 1D). TFEB, as a critical transcription factor regulating lysosomal biogenesis and lysosomal degradative pathway, is demonstrated to be involved in the pathogenesis of neurodegenerative diseases. Recent studies revealed that TFEB could facilitate oligomeric sAβ clearance by enhancing astrocytic lysosomal biogenesis (Xiao et al., 2014). To examine whether TFEB has an effect on microglial degradation of fAβ, we first exogenously expressed TFEB in BV2 cells and primary microglia by using lentiviral system. We observed less intracellular fAβ remained in the TFEB infected cells than that in the GFP infected cells at 18 h, indicating an enhancement of microglial degradation of fAβ. Meanwhile, microglial phagocytosis remains the same as intracellular fAβ levels at 3 h are comparable between cells infected with TFEB or GFP (Fig. 1E and 1G). Consistent with the gain-of-function data, siRNA specific knockdown of TFEB in microglia greatly reduce their capabilities to degrade fAβ (Fig. 1F and 1H). Intriguingly, we observed that TFEB has a tendency to translocate into nucleus upon stimulation of fAβ which is coincided with previous reports that TFEB will be activated under certain cellular stress (Figs. 2 and S2A). However, we proved that fAβ stimulation failed to inhibit mTORC1 activity which was previously reported to facilitate TFEB nuclear translocation (Fig. S2B), for the reason that fAβ stimulation could not inhibit the phorsphorylation status at specific sites of mTORC1 substrates as compared with the obvious inhibitory effects induced by mTORC1 inhibitor Torin1. Taken together, these data demonstrate that TFEB translocates into nucleus by fAβ stimulation in a mTORC1-independent pathway and facilitates fAβ degradation in microglia.

### TFEB is acetylated at lysine 116

**Figure 3:**
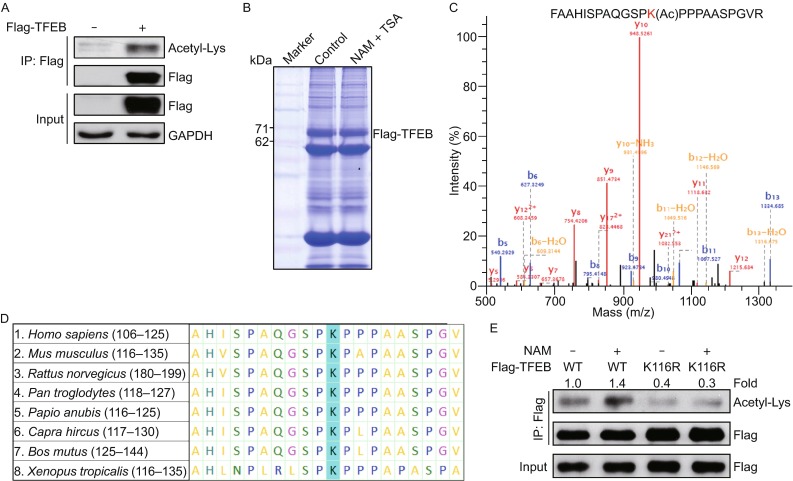
TFEB is acetylated at lysine 116. (A) Exogenous expressed TFEB is acetylated. Empty vector or Flag-tagged TFEB was transfected into HEK293T cells for 24 h treated with sirtuins inhibitor NAM (10 mmol/L) and HDAC I, II, and IV inhibitor TSA (0.5 μmol/L) and was immunoprecipitated for acetylation Western blotting analysis with the antibody against acetylated lysine. (B) Immunoprecipitated Flag-tagged TFEB for mass spectrometry analysis. Flagged-tagged TFEB was transfected into HEK293T cells for 24 h treated with or without NAM (10 mmol/L) and TSA (0.5 μmol/L) and was immunoprecipitated with anti-flag affinity agrose beads, followed by Commassie Blue R250 staining. (C) Identification of TFEB K116 acetylation using mass spectrometry analysis. Flagged-tagged TFEB was purified as depicted in (B) and then analyzed using mass spectrometry. (D) Alignment of the protein sequences of TFEB homologues in different species. The blue mark indicates the identified acetylated lysine residues of TFEB. (E) Deacetylation mimic of TFEB (K116R) counteracts NAM acetylation effects on TFEB. Flag-tagged TFEB (WT or K116R) were transfected into HEK293T cells for 24 h with or without additional 12 h treatment of NAM (10 mmol/L). Values were expressed as fold changes relative to TFEB-WT without NAM treatment and normalized to IP-Flag

A wide range of acetylated proteins have been identified using mass spectrometry, including numerous transcription factors whose activities are proved to be regulated by acetylation. To investigate acetylation status of TFEB, Flag-tagged human TFEB was transiently transfected into human embryonic kidney (HEK) 293T cells and its acetylation level was detected by an anti-acetylated lysine antibody. We observed a presence of acetylation on this exogenously expressed TFEB when the cells were treated with nicotinamide (NAM) that is an inhibitor of sirtuins family as well as trichostatin A (TSA) which inhibits activity of histone deacetylase (HDAC) classes I, II, and IV (Fig. 3A). This result prompted us to explore the potential acetylation sites on TFEB by the means of mass spectrometry analysis of TFEB purified from 293T cells treated with or without NAM and TSA (Fig. 3B). We repeatly identified an acetyl-lysine-containing peptide (FAAHISPAQGSPKPPPAASPGVR) that is matched with a region containing K116 on immunoprecipitated TFEB, in the cells treated with NAM and TSA (Fig. 3C). By performing sequence alignment of TFEB homologues from different species, we found that K116 is highly conserved in evolution (Fig. 3D). To further ascertain whether K116 site is acetylated, we generated single acetylation-deficient TFEB mutant (K-to-R mutation) by site-directed mutagenesis. As we expected, in contrast with wild-type TFEB (TFEB-WT), acetylation level of K116R mutant (TFEB-K116R) can no longer be elevated by NAM treatment (Fig. 3E). These data document that TFEB can be acetylated at K116 site which has not been previously recognized.

### TFEB is a deacetylation substrate of SIRT1

**Figure 4:**
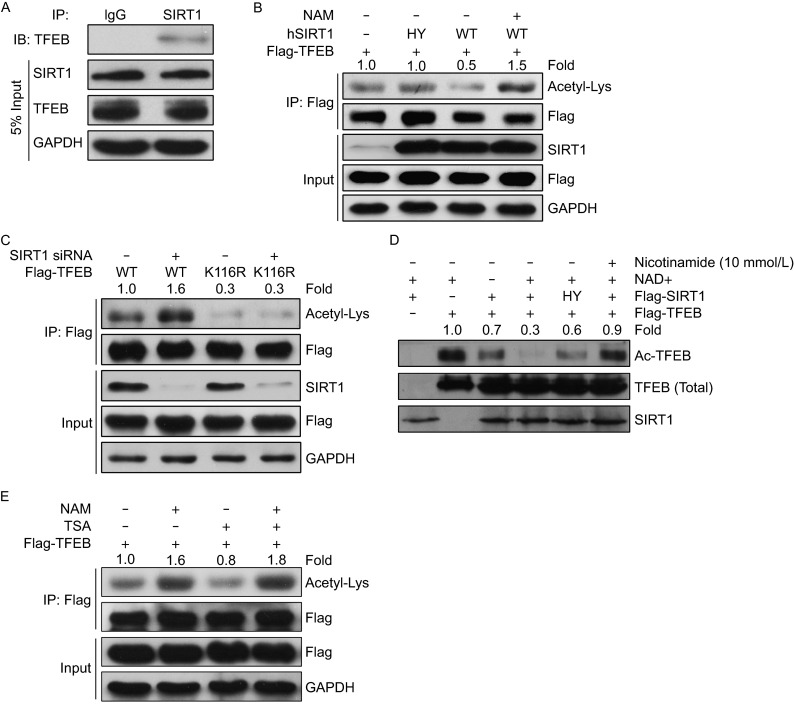
TFEB is a deacetylation substrate of SIRT1. (A) TFEB interacts with SIRT1. Endogenous coimmunoprcipitation assay was conducted with SIRT1 antibody and endogenous TFEB was detected by its specific antibody. (B) Catalytic activity of SIRT1 is required for deacetylation of TFEB. Flag-tagged TFEB was co-transfected into HEK293T cells with wild-type SIRT1 (WT) or its catalytically inactive mutant H363Y (HY), followed by additional treatment with or without NAM before harvest. Acetylation levels of TFEB were measured by IP Western blotting analysis. Values were expressed as fold changes relative to TFEB-WT without SIRT1 overexpression and normalized to IP-Flag. (C) Deacetylation mimic of TFEB (K116R) counteracts SIRT1 knockdown effects on acetylation of TFEB. Flag-tagged TFEB (WT or K116R) were co-transfected into HEK293T cells for 24 h with Scramble or SIRT1 siRNA. Acetylation levels of TFEB were measured by IP Western blotting analysis. Values were expressed as fold changes relative to TFEB-WT with scramble siRNA transfection and normalized to IP-Flag. (D) *In vitro* TFEB deacetylation by SIRT1. Purified Flag-TFEB and Flag SIRT1 (WT or HY) were co-incubated at 30°C for 6 h treated with or without NAD (5 mmol/L) or NAM (10 mmol/L). Acetylation levels of purified Flag-TFEB were measured by Western blotting analysis. Values were expressed as fold changes relative to TFEB-WT with NAD treatment and without SIRT overexpression and normalized to purified Flag-TFEB. (E) NAM, not TSA, increases TFEB acetylation. Flag-tagged TFEB was transfected into HEK293T cells treated with or without NAM (10 mmol/L) or TSA (0.5 μmol/L). Acetylation levels of TFEB were measured by IP Western blotting analysis. Values were expressed as fold changes relative to TFEB-WT without NAM or TSA treatment and normalized to IP-Flag

Given the evidence that TFEB can be acetylated at K116 site and the knowledge that acetylation status is regarded as a critical mechanism underlying regulation of activities of numerous transcription factors, we intended to identify the upstream regulator that targets TFEB. By performing co-immunoprecipitation (Co-IP) of exogenously expressed TFEB from 293T cells followed by mass spectrometry analysis, we detected SIRT1 as a potential candidate. To verify this result, we first overexpressed TFEB or SIRT1 in 293T cells and found endogenous SIRT1 (Fig. S3A) or TFEB (Fig. S3B) in the immunoprecipitates, respectively. Then we performed endogenous Co-IP with SIRT1 antibody and also detected TFEB in the immunoprecipitates (Fig. 4A), indicating that TFEB physically interacts with SIRT1. Next, we wondered whether SIRT1 could change the acetylation levels on TFEB. Treating 293T cells with resveratrol, a chemical activator of SIRT1, resulted in a reduction of acetylation on the immunoprecipitated TFEB, while its acetylation level was augmented by NAM treatment (Fig. S3C). In addition, the acetylation level on Flag-tagged TFEB was much lower in cells coexpressing wild-type SIRT1 (SIRT1-WT) than in cells expressing a catalytically inactive mutant SIRT1 (SIRT1-HY) (Fig. 4B). In parallel with these results, cells deficient of SIRT1 by knocking down with specific siRNA exhibited a higher level of acetylation on exogenously expressed TFEB-WT, but its deacetylation mimic TFEB-K116R counteracted SIRT1 knockdown effects, indicating K116 would be the site deacetylated by SIRT1 (Fig. 4C). To further ascertain whether TFEB is a substrate of SIRT1, we carried out *in vitro* deacetylation assay with purified SIRT1 and acetylated Flag-tagged TFEB (Fig. S3E). The data showed that SIRT1-WT, but not the catalytically inactive mutant SIRT1-HY, deacetylates TFEB in a NAD^+^-dependent manner, while treatment with SIRT1 inhibitor NAM reversed the effect of SIRT1 on TFEB (Fig. 4D). Despite these intriguing data, we intended to examine whether other HDAC families are involved in deacetylation of TFEB by using deacetylase inhibitors NAM and TSA. SIRT1 inhibitor NAM exerted a greater impact on increases of acetylation status of TFEB than HDACI/II/IV inhibitors TSA, indicating that TFEB is specifically acetylated by SIRT1 rather than other HDACs (Fig. 4E). Furthermore, SIRT6 and SIRT7 could not reduce acetylation levels on TFEB as compared with SIRT1 (Fig. S3D), excluding the possibility that SIRT6 or SIRT7 might involve in deacetylation of TFEB in the nucleus. Collectively, these findings demonstrate that SIRT1 is the primary deacetylase of TFEB *in vivo* and *in vitro*.

### SIRT1 accelerates fAβ degradation in microglia by enhancing lysosomal biogenesis

**Figure 5:**
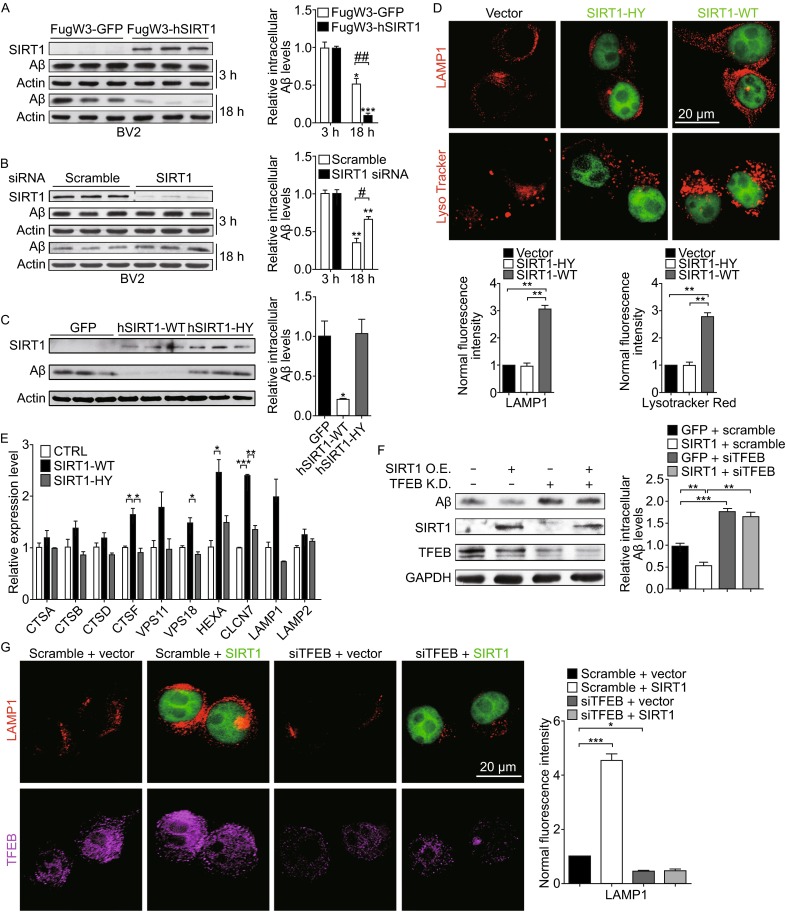
SIRT1 accelerates fAβ degradation in microglia by enhancing lysosomal biogenesis. (A and B) SIRT1 accelerates fAβ degradation in microglia. GFP or human SIRT1 (SIRT1) was overexpressed in BV2 cells by lentiviral system (A). Scramble or SIRT1 siRNA was delivered into BV2 cells for 72 h (B). Then the cells were incubated with fAβ (500 nmol/L) for 3 h or 18 h and were harvested for detection of intracellular Aβ levels by Western blotting analysis. Two-way ANOVA, comparison between different time points, *P < 0.05; **P < 0.01; ***P < 0.001. Unpaired Student’s *t*-test, comparison against the Fugw3-GFP or the Scramble, #*P* < 0.05, ##*P* < 0.01. (C) Catalytic activity of SIRT1 is required for degradation of fAβ. GFP or SIRT1 (WT or H363Y) was overexpressed in BV2 cells by lentiviral system. The cells were incubated with fAβ (500 nmol/L) for 18 h and were harvested for detection of intracellular Aβ levels by Western blotting analysis. One-way ANOVA with Turkey’s test, comparison against GFP, *P < 0.05. (D) SIRT1 stimulates lysosome biogenesis in microglia. Empty vector or GFP-tagged SIRT1 (WT or HY) was overexpressed in BV2 cells, followed by staining with an antibody against LAMP1 (Red) or LysoTracker Red, respectively. The fluorescence signal of each cell was estimated by examining more than 50 cells. One-way ANOVA with Turkey’s test, **P < 0.01; (E) SIRT1 upregulates TFEB induction of its target genes. Quantitative PCR (qPCR) analysis of TFEB target genes in BV2 cells overexpressed with empty vector as control or SIRT1 (WT or HY). Values represent mean ± SEM of three independent experiments. One-way ANOVA with Turkey’s test, comparison against CTRL, *P < 0.05; **P < 0.01; ***P < 0.001. (F) TFEB is required for SIRT1’s effects on accelerating fAβ degradation. BV2 cells were infected with lentiviral FugW3-GFP or FugW3-SIRT1 and were simultaneously transfected with scramble or TFEB siRNA for 72 h. The cells were incubated with fAβ (500 nmol/L) for an additional 18 h and were harvested for detection of intracellular Aβ levels by Western blotting analysis. One-way ANOVA with Turkey’s test, comparison against GFP with scramble siRNA, **P < 0.01; ***P < 0.001. (G) TFEB is required for SIRT1’s stimulation of lysosomal biogenesis. BV2 cells were transfected with scramble or TFEB siRNA for 72 h and were transfected with empty vector or GFP-tagged SIRT1-WT, followed by staining with an antibody against LAMP1 (Red). The fluorescence signal of each cell was estimated by examining more than 50 cells. One-way ANOVA with Turkey’s test, *P < 0.05; ***P < 0.001

Based on our evidence that TFEB can be deacetylated by SIRT1, we hypothesized that SIRT1 may increase lysosomal biogenesis by activating TFEB, which probably facilitates fAβ degradation in microglia. For this purpose, we first infected BV2 cells and primary microglia with lentivirally expressed SIRT1-WT as well as its catalytically inactive mutant SIRT1-HY. The intracellular Aβ levels reduced more dramatically after 18-h-incubation with microglia overexpressing SIRT1-WT than those overexpressing GFP as a vector control or SIRT1-HY (Figs. 5A, 5C, and S4A), indicating that only the catalytically active SIRT1 is able to facilitate microglial degradation of fAβ efficiently. Consistent with this result, siRNA-mediated SIRT1 deficiency in microglia attenuated the degradation of fAβ in both BV2 cells and primary microglia (Figs. 5B and S4B). In addition, pretreating microglia with resveratrol, the agonist of SIRT1, increased the clearance of intracellular fAβ in a dose-dependent manner (Fig. S4C), while SIRT1 inhibitor NAM abolished its capability to degrade fAβ (Fig. S4D). Meanwhile, we found that TSA could not affect microglial degradative capability on fAβ (Fig. S4E), indicating that it is SIRT1, but not other HDACs, that is responsible for fAβ degradation in microglia. These results prompted us to further examine whether SIRT1 could enhance lysosomal biogenesis. In BV2 microglia, we observed an expansion in abundance of lysosomal compartment stained with LysoTracker Red or lysosome membrane marker LAMP1 when exogenously transfecting with GFP-tagged SIRT1-WT, as compared with vector control or GFP-tagged SIRT1-HY (Fig. 5D). This result is in accordance with that in HeLa cells (Fig. S6A). The number of lysosomes, however, decreased in microglia with SIRT1 knockdown (Fig. S4G). Further, we observed microglia pretreated with NAM exhibited a diminished lysosomal biogenesis compared with the control, while TSA-treated microglia changed little (Fig. S4F). In addition, qPCR test revealed that exogenously overexpressed SIRT1-WT is able to stimulate expression of TFEB downstream targets which are involved in the process of lysosomal biogenesis as well as lysosome-mediated degradative pathways (Fig. 5E), while SIRT1 knockdown decreases expression of these genes (Fig. S4H). Given these evidence, we determined to investigate whether the function of SIRT1 in fAβ clearance in microglia depends on its deacetylation on TFEB. Simultaneously overexpressing SIRT1 by lentivirus and knocking down endogenous TFEB neither promote fAβ degradation nor enrich the abundance of lysosomes in BV2 microglia, indicating TFEB is required for SIRT1’s effects on facilitating fAβ degredation and lysosomal biogenesis in microglia (Fig. 5F and 5G). Collectively, these evidence suggest that SIRT1 accelerates fAβ degradation by enhancing lysosomal biogenesis in microglia.

### Deacetylation status at K116 of TFEB stimulates fAβ degradation by facilitating lysosomal biogenesis

**Figure 6:**
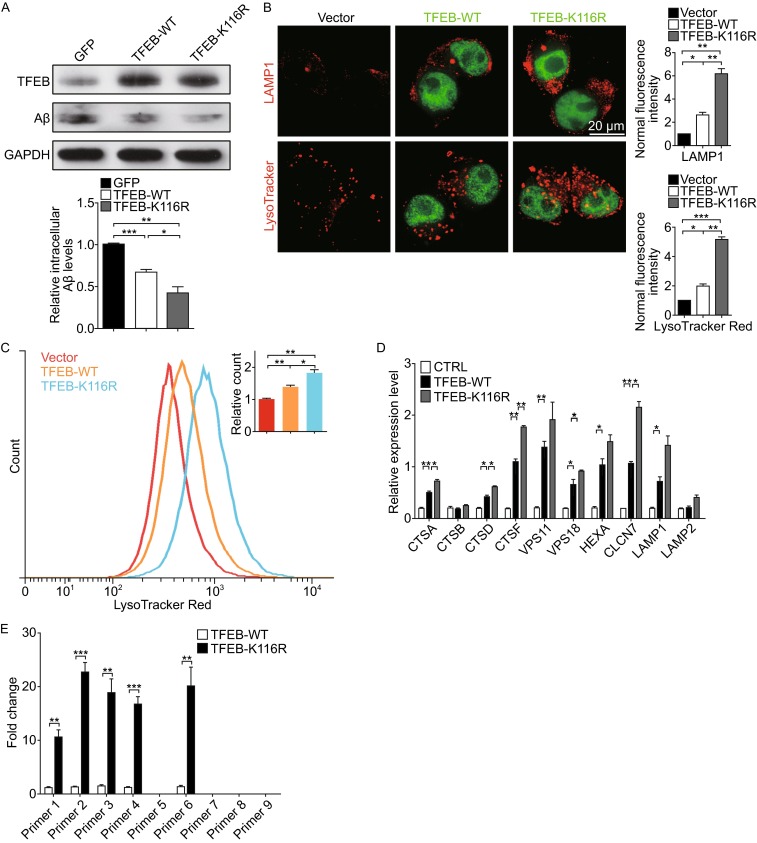
Deacetylated TFEB facilitates fAβ degradation by enhancing lysosomal biogenesis. (A) Deacetylation of TFEB at K116 futher facilitates fAβ degradation. BV2 cells were infected with lentivirally overexpressed FugW3-GFP or FugW3-TFEB (WT or K116R). The cells were incubated with fAβ (500 nmol/L) for an additional 18 h and were harvested for detection of intracellular Aβ levels by Western blotting analysis. The band intensity was measured in three independent experiments indicating relative intracellular Aβ levels and the mean ± SEM. are shown in the right panel. One-way ANOVA with Turkey’s test, *P < 0.05; **P < 0.01; ***P < 0.001. (B) Deacetylation of TFEB at K116 further stimulates lysosomal biogenesis in microglia. Empty vector or GFP-tagged TFEB (WT or K116R) was overexpressed in BV2 cells, followed by staining with an antibody against LAMP1 (Red) or LysoTracker Red, respectively. The fluorescence signal of each cell was estimated by examining more than 50 cells. One-way ANOVA with Turkey’s test, *P < 0.05; **P < 0.01; ***P < 0.001. (C) Flow cytometric analysis of lyososomes stained with LysoTracker Red in BV2 cells treated as in (B). Values of mean fluorescence were expressed as fold changes. One-way ANOVA with Turkey’s test, *P < 0.05; **P < 0.01. (D) Deacetylation status at K116 further upregulates expression of TFEB target genes. qPCR analysis of TFEB target genes in BV2 cells overexpressed with empty vector as control or TFEB (WT or K116R). Values represent mean ± SEM of three independent experiments. One-way ANOVA with Turkey’s test, comparison against CTRL, *P < 0.05; **P < 0.01. (E) ChIP-qPCR analysis for TFEB binding to its target gene *CLCN7*. The histogram shows the amount of immunoprecipitated DNA as detected by qPCR assay. Values were normalized to the input and displayed as relative enrichment over a mock control. Values represent mean ± SEM of three independent experiments. One-way ANOVA with Turkey’s test, **P < 0.01; ***P < 0.001

Given our previous observations that SIRT1 is the primary deacetylase of TFEB and its enhancement of fAβ degradation in microglia counts on TFEB, we determined to explore the possibility that deacetylated TFEB might increase lysosomal function and facilitate fAβ degradation. To address this question, we infected BV2 microglia with lentivirally overexpressed TFEB-WT or TFEB-K116R. The result noted that K116R mutant displayed a stronger capability in degradation of fAβ than its wild-type (Fig. 6A), which is accompanied by observations that more lysosomal compartments were induced in microglia and HeLa cells transfected with TFEB-K116R than TFEB-WT (Figs. 6B and S6B). Accordingly, analysis of these acidic organelles by fluorescence activated cell sorting (FACS) stained with LysoTracker Red revealed that there was an expansion of the quantity of lysosomes in the microglia infected with lentivirally-expressed TFEB-K116R (Fig. 6C). Furthermore, transcription of TFEB downstream targets was significantly upregulated in the presence of TFEB-K116R mutant (Fig. 6D). In order to confirm that the deacetylated K116R mutant promotes TFEB binding to its target genes, we performed chromatin immunoprecipitation (ChIP) assay and found that TFEB-K116R had a much stronger binding in the promoter of TFEB target gene *CLCN7* than the TFEB-WT (Fig. 6E). To further confirm it is the deacetylation status of TFEB that facilitates fAβ degradation by upregulating lysosomal function in microglia, we also evaluated the effects of TFEB-K116Q mutant (acetylation mimic). The results indicated that microglia with TFEB-K116Q overexpression could neither facilitate fAβ degradation (Fig. S5B) nor upregulate the lysosomal biogenesis and function (Fig. S5C and S5D). Collectively, these data indicate that deacetylation of TFEB intensifies its contribution to microglial degradation of fAβ.

### Microglial expression of deacetylated TFEB reduces amyloid plaques in brain slices from APP/PS1 mice

**Figure 7:**
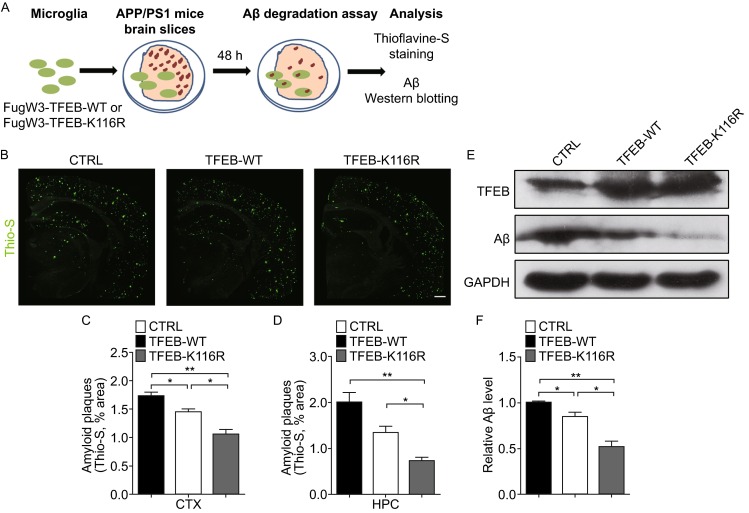
Microglial expression of deacetylated TFEB reduces amyloid plaques in the brain slices from APP/PS1 mice. (A) *Ex vivo* degradation assay. BV2 microglia were infected with lentivirally overexpressed FugW3-TFEB (WT or K116R) and co-cultured with 10 μm thick freshly cut, unfixed brain slices from aged APP/PS1 transgenic mice. After 48 h the slices were used for subsequent analysis. (B–E) Deacetylation of TFEB at K116 further reduces amyloid plaques. BV2 microglia overexpressed with vector control (CTRL) or TFEB (WT or K116R) were co-cultured with brain sections prepared as depicted in (A), followed by staining amyloid plaques with thioflavine-S (Green) (B–D) or by Western blotting analysis of fAβ levels (E and F). CTX, cortex; HPC, hippocampi. Results are mean area fraction of amyloid plaques ± SEM for 3 mice per group. The band intensity was measured in three independent experiments indicating relative fAβ levels and the mean ± SEM. One-way ANOVA with Turkey’s test, *P < 0.05; **P < 0.01. Scale bar, 200 μm

**Figure 8:**
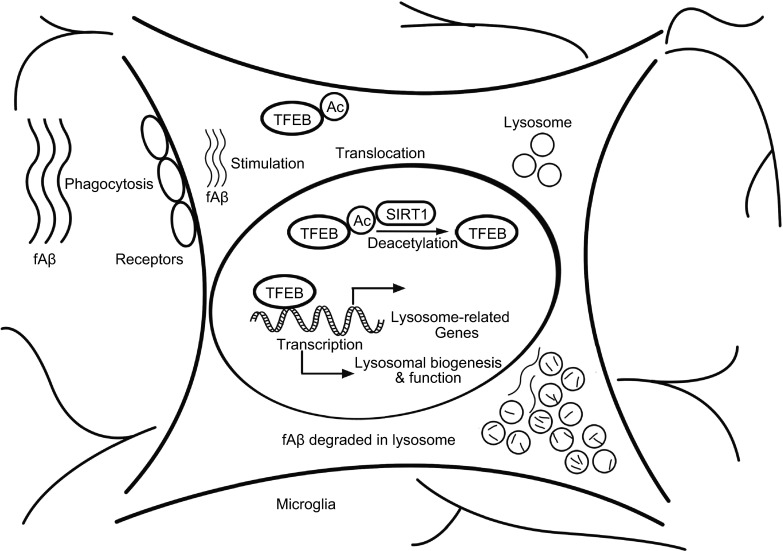
A schematic model of TFEB deacetylation on facilitating fAβ degradation in microglia by enhancing lysosomal biogenesis. In the presence of fAβ stimulation, microglia take up fAβ and transport them into lysosomes. Meanwhile, TFEB translocates into nucleus where it interacts with SIRT1 and is deacetylated at K116. Deacetylation of TFEB enhances its transcriptional induction of its downstream target genes closely associated with lysomomal biogenesis, which eventually upregulates microglial capability in degradation of fAβ

To evaluate whether deacetylated TFEB could enhance microglial degradation of amyloid plaques, we performed *ex vivo* brain slice assay as described previously (Bard et al., 2000), where microglial cells were co-cultured with brain slices from aged APP/PS1 transgenic mice (Fig. 7A). Quantitative analysis of thioflavine-S staining indicated that BV2 microglia lentivirally overexpressed with TFEB-K116R were significantly more efficient at clearing the aggregates deposited in the cortex (Fig. 7B and 7C) and hippocampus (Fig. 7D) when compared with its wild-type. In addition, independent Western blotting measurements of Aβ levels in the brain slices achieved a similar result (Fig. 7E and 7F). These *ex vivo* findings were consistent with the enhancement of microglial degradation of fAβ *in vitro*, suggesting a strengthened capability of microglial removal of Aβ aggregates which is triggered by deacetylation of TFEB. According to our results, deacetylation of TFEB, carried out by its deacetylase SIRT1, promotes microglial degradation of fAβ and amyloid plaques by upregulating lysosomal function and biogenesis, which highlights acetylation status of TFEB as a critical role in the degradative pathway in microglia (Fig. 8).

## Discussion

Microglia are the principle immune phagocytic cells in the CNS which constantly survey their adjacent environment for maintainance of the homeostasis within the brain. Previous work revealed that functional deficiency in microglia is associated with much more severe deposition of amyloid plaques in AD mouse models, indicating a protective role of microglia in AD (Zhang et al., 2007). Indeed, we and others have observed that microglia internalize Aβ through membrane receptors and transport them into lysosomes for degradation (Doens and Fernandez, 2014; Ma et al., 2013). In this paper, we demonstrated that the ability of microglia to degrade fAβ could be facilitated by TFEB-mediated upregulation of intracellular degradative systems.

Several lines of evidence pointed out that the amyloid deposits cannot be fully eliminated or even remain stable albeit by surrounding coverage of activated microglia (Bolmont et al., 2008; Wegiel et al., 2001), which may be explained in part by the dysfunction of lysosomes in the presence of Aβ stimulation (Hickman et al., 2008; Majumdar et al., 2007). Subsequent studies further clarified that attempts to upregulating intracellular lysosomal hydrolytic enzymes or stabilizing a fitting lysosomal acidic environment eventually enhance or restore microglial capability to degrade Aβ (Majumdar et al., 2011; Majumdar et al., 2008), which may be suggestive of strengthening lysosomal function as a novel approach to increase Aβ clearance. In this regard, our results validated that TFEB, the major regulator of lysosomal biogenesis, facilitates or rejuvenates microglial capability in degradation of fAβ and amyloid plaques by upregulating lysosomal quantity and enhancing lysosomal function.

Emerging evidence pointed out that defective activation of TFEB probably underlies aberrant lysosomal function failing to degrade aggregated toxic proteins in prevalent neurodegenerative diseases (Decressac et al., 2013; Tsunemi et al., 2012; Xiao et al., 2014). Therefore, appropriate regulation of TFEB activity has attracted more and more attention in countering the pathogenesis of these diseases (Settembre et al., 2013b). Despite the fact that TFEB can be activated by its own stimulation in a transcriptional autoregulatory pathway (Settembre et al., 2013a), post-translational modification play an essential role in modulating TFEB activity, among which phosphorylation is the first to be studied. Under basal conditions, TFEB is mainly sequestered in cytosol where it remains to be phosphorylated by rapamycin complex mTORC1 (Settembre et al., 2011). However, once upon specific conditions such as starvation or mTOR suppression, TFEB is rapidly dephosphorylated and translocates into nucleus for transcriptional activation of lysosomal system, a process proved to be dependent on autophagosome-lysosome fusion (Zhou et al., 2013). Nevertheless, it still remains a mystery that whether TFEB transcriptional activity needs proper regulation within the nucleus and could other modifications be involved in such regulatory mechanisms. In this regard, for the first time, we identified acetylation as a prominent post-translational modification in regulating TFEB transcriptional activity within the nucleus. Our results indicated that TFEB translocates into nucleus in a mTORC1-independent pathway once microglia are under exposure to fAβ. Although further investigation is needed to unveil the mechnisms underlying this observation, our findings suggest that such translocation may enable TFEB to be deacetylated by nuclear-located SIRT1, which enhances its transcriptional induction of target genes closely associated with lysosomal biogenesis, ultimately resulting in a robust degradation of intracellular fAβ.

Protein acetylation has been recently recognized as a significant approach in controlling autophagic processes such as elimination of damaged organelles or toxic protein aggregates (Banreti et al., 2013). Previous studies revealed that acetylation of ATG proteins, the autophagy core components, by acetyltransferase p300 results in autophagy inhibition (Lee and Finkel, 2009), whereas this effect can be reversed by NAD^+^-dependent deacetylase SIRT1 under particular cellular stresses (Lee et al., 2008). In addition, SIRT1-mediated deacetylation of FOXO transcription factors is able to activate autophagy by upregulation of core autophagy genes in the process of autophagosome formation (Chakrabarti et al., 2011; Zhao et al., 2007). In the present study, we demonstrated that deacetylation of TFEB by SIRT1 facilitates lysosomal biogenesis, which suggests a novel mechanism regulating intracellular degradative pathways and validating the importance of protein acetylation in these processes. However, the exact regulatory role of protein acetylation in autophagy-lysosome system still needs to be defined.

SIRT1, the well-studied NAD^+^-dependent deacetylase, has been realized as a master regulator of multiple cellular metabolic processes, including various common neurodegenerative disorders. In particular, AD transgenic mouse models displayed a strikingly reduced Aβ burden in the hippocampus and cortex when SIRT1 was chemical activated or overexpressed by lentiviral infection (Karuppagounder et al., 2009). The underlying mechanism was further explained by observations that neuronal SIRT1 promotes the nonamyloidogenic APP-processing pathway either by directly enhancing α-secretase activity or by upregulating expression of α-secretase, respectively (Donmez et al., 2010; Qin et al., 2006). In contrast with the knowledge that SIRT1 prevents neuronal Aβ production, little is known about SIRT1’s function in clearance of Aβ. Previous studies suggested that SIRT1 in microglia contributes to amelioration of Aβ-induced inflammatory response by suppressing NF-κB and IL-1β signaling pathway (Chen et al., 2005; Cho et al., 2015). In this study, we found that SIRT1 is able to regulate microglial degradation of fAβ. Moreover, we identified TFEB as a novel substrate of SIRT1 and provided evidence that SIRT1 upregulates lysosomal biogenesis by stimulating TFEB induction of its downstream targets, ultimately resulting in increased fAβ clearance in microglia. However, considering about the fact that several substrates of SIRT1 were reported to positively regulate autophagy such as Foxo3 and Atg proteins (Lee and Finkel, 2009; Zhao et al., 2007), our present data cannot exclude the possibility that SIRT1 may promote fAβ degradation by regulating other molecules in lysosomal-autophagy degradative pathway. Further investigation is needed to achieve a better understanding of the underlying mechanisms of SIRT1-mediated fAβ degradation. Overall, our findings uncover a novel mechanism that SIRT1 protects against Aβ deposits by facilitating microglial clearance of Aβ other than preventing neuronal production of Aβ.

It is noteworthy that our results are in accordance with a recent work indicating that TFEB accelerates astrocytic capability in Aβ removal (Xiao et al., 2014). In fact, these two glia cells may play distinct roles in Aβ clearance depending on amyloid aggregation states and pathogenesis stages. Astrocytes possibly play a constitutive role in the very early AD stages by taking up oligomeric sAβ, while microglia mainly function in the late stages as fAβ accumulates to form fibrillar deposits (Condello et al., 2015; Mulder et al., 2014; Weldon et al., 1998). In fact, we did conduct the experiment by culturing sAβ with microglia in our degradative model and found that deacetylated TFEB could degrade sAβ more efficiently as well (Fig. S5A), which collectively verify the vital role of deacetylation status of TFEB in Aβ degradation. Moreover, TFEB overexpression in neuron is recently reported to reduce Aβ generation by enhancing APP degradation in lysosomes (Xiao et al., 2015). In this scenario, our results favour a plausible novel therapy that upregulation of TFEB activities in both glia cells and neuron may help to coordinately ameliorate AD pathogenesis, providing a better healing of AD.

In summary, we identify protein acetylation as a novel mechanism underlying regulation of TFEB transcriptional activity for the first time, and demonstrate that deacetylation of TFEB in microglia enhances lysosomal biogenesis by stimulating expression of its target genes, which ultimately facilitates fAβ degradation and decreased amyloid plaque deposition. Our findings highlight that TFEB is a central regulator in lysosome-mediated clearance of toxic protein aggregates. Also, distinct post-translational modifications of TFEB occurring at different cellular localization may regulate TFEB activity coordinately in the autophagy-lysosome pathway. These findings may provide an appealing avenue in countering pathogenesis of AD and other neurodegenerative diseases.

## Materials and methods

### Cell culture

BV2, HEK293T, and HeLa cell lines were obtained from Cell Resource Center of Peking Union Medical College Hospital and cultured in Dulbecco’s Modified Eagle Medium (DMEM) supplemented with 10% fetal bovine serum (FBS) (Hyclone, USA) and antibotics (100 U/mL penicillin, 100 µg/mL streptomycin). Cells were incubated at 37°C in a 95% humidified atmosphere with 5% CO_2_.

Primary microglia were prepared from the whole brains of 24-h-old Sprague-Dawley (SD) rats as described previously (Ma et al., 2013). Briefly, pups were decapitated and then the meninges and blood vessels were removed from the cortices which were minced and digested with Dnase I (0.01%) and trypsin (0.25%) for 30 min at 37°C. The digestion was stopped by DMEM containing 10% FBS and 1% penicillin-streptomycin. The cell mixture were then triturated and plated on T75 flask (Corning, USA). The media were changed to fresh DMEM with 10% FBS 24 h later and were cultured for another 14–21 days at 37°C. Primary microglia were isolated by shaking the plates for 2 h at 260 rpm. Cells were then counted and plated for experiments at the appropriate density in DMEM/F12 containing 2% FBS. The media were changed to serum-free DMEM/F12 overnight before conducting the experiment. The enriched microglia were >98% pure as determined by immunoflurescence shown with Iba1-immunoreactive (IR) and GFAP-IR cells (Fig. S1C).

### Preparation of fibrillar Aβ

Human Aβ_1–42_ (20276; AnaSpec, USA) or Hilyte-Fluor-488 fibrillar Aβ_1–42_ (60479; AnaSpec, USA) were dissolved in 1 mmol/L phosphate-buffered saline buffer (PBS) and incubated at 37°C for 7 days and stored at −20°C before use. The ultra structure of the peptide aggregates was examined by transmission electron microscopy.

### Plasmids and transfection

Human TFEB was subcloned into pCMV-Tag2B and pEGFP-N3, respectively. Human SIRT1-WT and SIRT1-HY was subcloned into pCMV-3×HA and pEGFP-N3, respectively. TFEB-K116R was mutated from TFEB- pCMV-Tag2B and TFEB-pEGFP-N3, respectively. Transient transfection of plasmids were conducted using Lipofectamine LTX according to the instruction (Invitrogen), followed by changing the medium 4 h later. The cells were harvested 24 h later for the subsequent experiments.

### Lentivirus infection

The FugW3 four-plasmid-lentivirus system were utilized to overexpress SIRT1 (WT or HY) and TFEB (WT or K116R) in BV2 and primary microglia. FugW3-GFP as a vector control and FugW3-SIRT1-WT, -SIRT1-HY, -TFEB-WT, and -TFEB-K116R constructs were transfected into 293T-packaging cells with three other helper plasmids (RRE, REV, and VsVg) respectively, using Lipofectamine LTX. Medium was changed 12 h later for another 60 h incubation before collecting the supernatant which was filtered through a 0.45 μm filter, aliquated and stored at −80°C. BV2 or primary microglia was infected with the supernatant in the presence of 2 µg/mL Polybrene for 24 h before changing the medium. Cells were harvested 48–72 h later for the subsequent experiments.

### RNA interference

BV2 or primary microglia in 12-well plates were transfected with siRNAs against target genes (SIRT1 siRNA: sc-40987; TFEB siRNA: sc-38510; Santa Cruz Biotechnology, CA) or scramble siRNA as control with Lipofectamine RNAiMAX (Invitrogen, Carlsbad, CA). Cells were harvested 48–72 h later for the subsequent experiments.

### fAβ uptake and degradation

Measurements of fAβ uptake and degradation were performed as depicted previously (Jiang et al., 2008). Briefly, to measure localization of the internalized fAβ, BV2 cells or primary microglia were plated in 12-well plates, and 500 nmol/L Hilyte-Fluor-488 fibrillar Aβ_1–42_ was added for 30 min. The cells were stained with LysoTracker Red before confocal imaging.

To analyse microglial degradation, 500 nmol/L *in vitro* synthetic fAβ_1–42_ was incubated with BV2 and primary microglia for varying times. Cells were infected with lentivirally overexpressed GFP, SIRT1 (WT or HY), TFEB (WT or K116R) or were transfected with siRNA for SIRT1 or TFEB for 72 h before incubation with 500 nmol/L fAβ_1–42_ for another 3 h or 18 h, indicating microglial phagocytosis or degradation of fAβ, respectively. Subsequently, cells were thoroughly washed with DMEM three times and lysed for Western blotting Analysis. The band intensity was measured in three independent experiments indicating relative intracellular fAβ levels. Inhibitors for lysosome or NEP were added to the cells 2 h before adding fAβ and were kept in the medium before harvest.

### Antibodies and reagents

Mouse monoclonal anti-β-actin (ab3280; Abcam, MA); mouse monoclonal anti-GAPDH (cw0266; CWBio; China); mouse monoclonal anti-HA (H9658; Sigma, St. Louis, MO); mouse monoclonal anti-Flag M2 antibody (F3165; Sigma, St. Louis, MO); mouse monoclonal anti-SIRT1 (8469; Cell Signaling Technology, MA); rabbit polyclonal anti-SIRT1 (2493; Cell Signaling Technology, MA); goat polyclonal anti-TFEB (ab2636; Abcam, MA); rabbit polyclonal anti-TFEB (sc48784; Santa Cruz Biotechnology, CA); mouse monoclonal anti-Aβ_1–16_ (6E10) (SIG39320, Covance, USA); rabbit monoclonal acetylated-lysine antibody (9814; Cell Signaling Technology, MA); rabbit polyclonal anti-Lamp1 (ab 24170; Abcam, MA); LysoTracker Red (C1046; Beyotime, China); Leupeptin (L2884; Sigma, St. Louis, MO); Chloroquine (C6628; Sigma, St. Louis, MO); Phosphoramidon (R7385; Sigma, St. Louis, MO); Thioflavine-S (T1892; Sigma, St. Louis, MO).

### Western blotting analysis

Cells were harvested and lysed with lysis buffer consisting of 50 mmol/L Tris-HCl (pH 7.5), 150 mmol/L NaCl, 1% NP-40, 1% SDS, protease inhibitors cocktail (Sigma, St. Louis, MA) and 10% the mixture of deoxyribonuclease I (1 mg/mL) and ribonuclease A (0.25 mg/mL) at 4°C. The lysates were sonicated, followed by centrifugation at 14,800 ×*g* at 4°C for 10 min to remove the insoluble substances. The protein concentration was determined by 2-D Quantitative Kit (GE Healthcare, USA). Equal amount the lysates were resolved by 10% SDS-PAGE and transferred onto PVDF membranes using a semi-dry blotting apparatus (Bio-Rad, USA). After blocking with 5% non-fat milk in phosphate buffered saline (PBS) with 0.05% Tween 20, the membranes were incubated overnight at 4°C with primary antibodies, followed by incubation with HRP-conjugated secondary antibodies (1031-05 or 4050-05; Southern Biotech, MA). Protein bands were detected by chemiluminescence (Millipore, Billerica, MA).

### *Ex vivo* degradation assay

*Ex vivo* degradation assays were performed as previous described (Bard et al., 2000). Briefly, 12-month-old APP/PS1 transgenic mice were perfused with saline and cryostat sections (10 μm in thickness) were cut from snap-frozened brain and mounted onto glass coverslips pre-coated with poly-L-lysine. Brain sections were dried for at least 2 h at room temperature and washed with hybridoma serum-free media (H-SFM; Thermo Fisher) containing 1% FBS and 1% penicillin/streptomycin. BV2 microglia were seeded (8 × 10^5^ cell/mL) and co-cultured with the brain sections in 24-well plates for 48 h at 37°C with 5% CO_2_. After incubation, sections were washed twice with PBS and fixed with 4% paraformaldehyde for subsequent thioflavine-S staining or lysed in 6.25 mol/L guanidinhydrochloride for Western blotting analysis.

### Immunoprecipitation

Cells were pretreated with TSA (0.5 μmol/L) and NAM (10 mmol/L), or seperately. Then cells were harvested and lysed in five volume of lysis buffer (25 mmol/L Tris-HCl, 150 mmol/L NaCl, 3 mmol/L MgCl_2_, 0.5% NP-40, 1 mmol/L DTT, 5% Glycerol, 1% PI, pH 7.5) for rotating at 4°C for 2 h, followed by centrifugation at 21,000 ×*g* for 30 min. Then the supernatant were mesured by 2-D Quantitative Kit. Equal amount of protein was incubated with the ANTI-FLAG M2 Affinity Gel (Sigma, St. Louis, MO) at 4°C overnight. Immunoprecipitates were washed with lysis buffer for four times and eluted with SDS loading buffer, followed by SDS-PAGE separation and Western blotting Analysis.

### Mass spectrometry analysis

HEK293T cells were transfected with Flag-tagged TFEB for 24 h, then the cells were pretreated with 0.5 μmol/L TSA and 10 mmol/L NAM for 12 h before harvest. Cells were lysed and immunoprecipitated as depicted above. The eluted mixture was resolved by 10% SDS-PAGE and stained with Commassie Blue R250 and the bands were excised from the gel, followed by tryptic digestion and mass spectrometry. Modification identification was measured with the database search accordingly and peptide identifications were validated with Proteome Discoverer 1.4.

### *In vitro* deacetylation assay

Flag-tagged SIRT1 (WT or HY) and TFEB were purified from 293T cells by immunoprecipitation. The deacetylation reaction was performed in 100 μL of reaction buffer containing 5 μg of puridied Flag-tagged TFEB proteins and 15 μg purified Flag-SIRT1-WT or SIRT1-HY proteins in Tris-HCl buffer (Tris-HCl, PH 8.5, 50 mmol/L; NaCl 50 mmol/L; MgCl_2_ 4 mmol/L; DTT 1 mmol/L; Glycerol 5%; NAD^+^ 5 mmol/L; NAM 10 mmol/L) at 30°C for 6 h. During the incubation, the buffer was mixed every 5 min and the reaction was stopped by SDS loading buffer, followed by SDS-PAGE separation and Western blotting Analysis.

### Immunofluorescence

Cells were 4% PFA fixed for 20 min, permeabilised in 0.1% PBST for 20 min, and then blocked in 10% BSA/0.05% PBST for 1 h at room temperature. The cells were then incubated with primary antibody in 1% BSA/PBST overnight at 4°C, followed by a further incubation at room temperature for 1 h with AlexaFluor secondary antibodies. Images were acquired with LSM 710 NLO & DuoScan System confocal laser-scanning microscope (Zeiss, Jena, Germany).

### Flow cytometric analysis

Cells were incubated with LysoTracker Red (0.1 μmol/L) for 10 min and washed by PBS thoroughly, followed by flow cytometric analysis on BD LSRFortessa instrument, 30,000 events per run. FlowJo software was used to perform data analysis.

### RNA isolation and quantitative real-time PCR

Total RNA was isolated from cells with different treatments by Trizol reagent (Invitrogen, USA), and 1 μg of total RNA was reverse transcribed to cDNA using HiFi-MMLV cDNA first strand synthesis Kit (CW Bio, China). Quantitative PCR (qPCR) was carried out by mixing cDNA with GoTaq qPCR Master Mix (Promega, USA) and the reaction was performed on the CFX96 Real Time PCR Detection system (Bio-Rad, USA), under the following conditions: 95°C, 2 min; 95°C, 15 s; 60°C, 30 s; 72°C, 25 s; followed by 40 cycles of 95°C, 15 s; 60°C, 2 min.

The primers for qPCR were designed according to gene sequences published in theGenBank (SIRT1, forward, 5′-TGTGTCATAGGTTAGGTGGTGA-3′, reverse, 5′-AGCCAATTCTTTTTGTGTTCGTG-3′; TFEB, forward, 5′-AAGGTTCGGGAGTATCTGTCTG-3′, reverse, 5′-GGGTTGGAGCTGATATGTAGCA-3′; CTSA, forward, 5′-TCCCAGCATGAACCTTCAGG-3′, reverse, 5′-AGTAGGCAAAGTAGACCAGGG-3′; CTSB, forward, 5′-ACAACGTGGACATGAGCTACT-3′, reverse, 5′-TCGGTAAACATAACTCTCTGGGG-3′; CTSD, forward, 5′-TGCTCAAGAACTACATGGACGC-3′, reverse, 5′-CGAAGACGACTGTGAAGCACT-3′; CTSF, forward, 5′-AGAGAGGCCCAATCTCCGT-3′, reverse, 5′-GCATGGTCAATGAGCCAAGG-3′; VPS11, forward, 5′-CAAGCCTACAAACTACGGGTG-3′, reverse, 5′-GAGTGCAGAGTGGATTGCCA-3′; VPS18, forward, 5′-CACTCGGGGTATGTGAATGCC-3′, reverse, 5′-TCGGAAGGGGTGAAGTCAATG-3′; HEXA, forward, 5′-ACGTCCTTTACCCGAACAACT-3′, reverse, 5′-CGAAAAGCAGGTCACGATAGC-3′; CLCN7, forward, 5′-CCACGTTCACCCTGAATTTTGT-3′, reverse, 5′-AAACCTTCCGAAGTTGATGAGG-3′; LAMP1, forward, 5′-CAGATGTGTTAGTGGCACCCA-3′, reverse, 5′-TTGGAAAGGTACGCCTGGATG-3′; LAMP2, forward, 5′-GCACAGTGAGCACAAATGAGT-3′, reverse, 5′-CAGTGGTGTGTATGGTGGGT-3′).

### Chromatin immunoprecipitation assay

Chromatin immunoprecipitation assay (ChIP) was performed as depicted previously (Hug et al., 2004). In brief, cells were transfected with Flag-tagged TFEB-WT or TFEB-K116R for 24 h. The cells were harvested for immunoprecipitation with ANTI-FLAG M2 Affinity Gel (Sigma, St. Louis, MO) and were then subjected to the ChIP assay following the manufacturer’s instruction of Chromatin immunoprecipitation kit (9002, Cell Signaling Technology, MA). qPCR was performed to figure out the TFEB binding in promoter. The data were analysed and displayed as described previously (Settembre et al., 2013a). Primer sequences of *CLCN7* for amplifying purified DNA were listed in the Table S1.

### Statistical analysis

All functional experiments were repeated at least in three independent experiments. The band intensity in Western blot analysis was quantified by ImageJ. Data were analyzed by GraphPad Prism 5.0 software and shown by the mean ± SEM. Multiple sets of data were analyzed by one-way or two-way ANOVA, and unpaired Student’s *t* test was used to analyze two sets of data. Significance level was set at *P* < 0.05.

## Electronic supplementary material

Below is the link to the electronic supplementary material.

Supplementary material 1 (PDF 935 kb)
