# Supplementary material for: Deacetylation of TFEB promotes fibrillar Aβ degradation by upregulating lysosomal biogenesis in microglia
Source: Protein Cell. 2016 May 21;7(6):417–33. doi: 10.1007/s13238-016-0269-2 (PMC4887328; doi:10.1007/s13238-016-0269-2)
Supplement: Supplementary file 1 — Supplementary material 1 (PDF 935 kb) [file 13238_2016_269_MOESM1_ESM.pdf]

Deacetylation of TFEB promotes fibrillar Aβ degradation by upregulating lysosomal biogenesis in microglia

Jintao Bao[1], Liangjun Zheng[1], Qi Zhang[1], Xinya Li[1], Xuefei Zhang[1], Zeyang Li[1], Xue Bai[1], Zhong Zhang[1], Wei Huo[1], Xuyang Zhao[2], Shujiang Shang[3], Qingsong Wang[1, *], Chen Zhang[3, *] and Jianguo Ji[1, *]

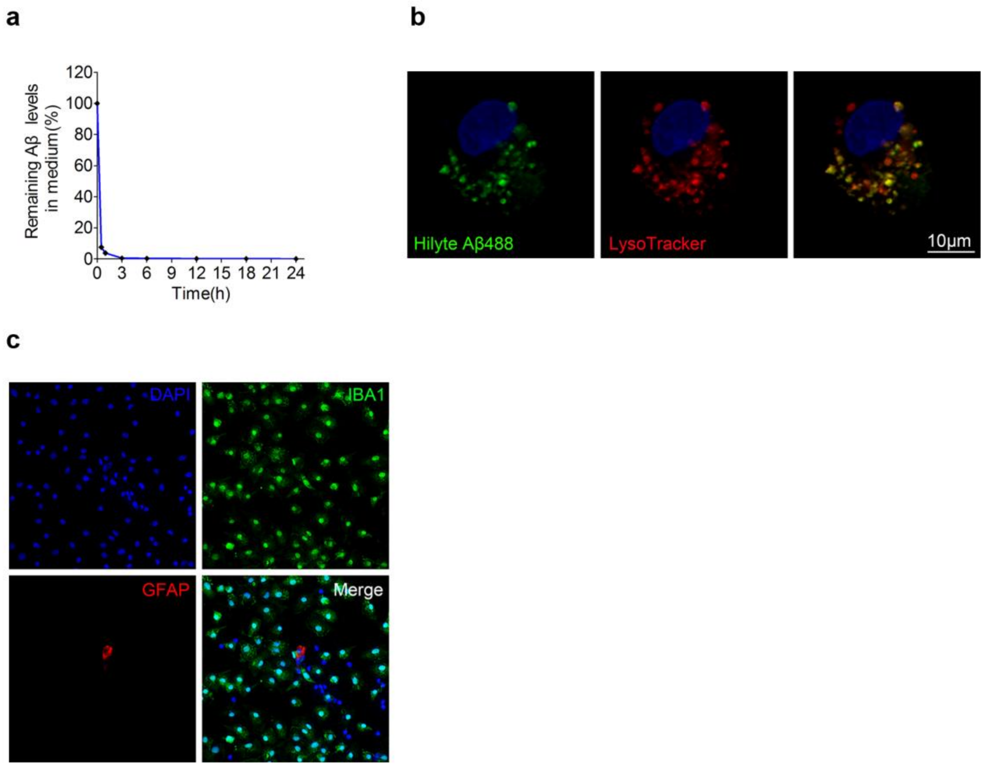

Supplementary Figure 1 | Fibrillar Aβ is degraded in microglial lysosomes. (**a**) Microglia efficiently internalize and degrade fAβ and no longer resecrete. BV2 cells were incubated with fAβ (500nM) at 37℃ and the fAβ levels in media were measured using ELISA for resecreted Aβ. (**b**) Fibrillar Aβ is rapidly trafficked into lysosomes. Confocal imaging of live primary microglia 30 min after addition of Hilyte488-labeled fAβ (500nM) showed localization of Aβ (Green) within lysosomes stained with LysoTracker (Red). (**c**) Identification of isolated primary microglia. The isolated primary microglia were immunostained with antibody specific to microglia, IBA1 (Green) or antibody specific to astrocytes, GFAP (Red).

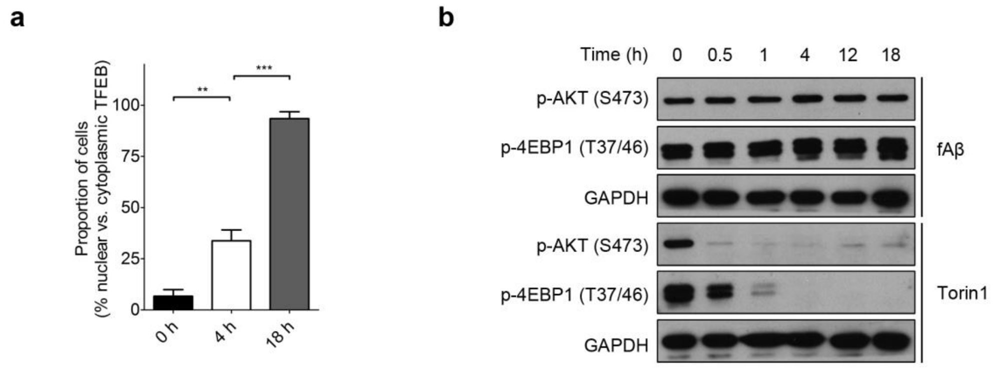

Supplementary Figure 2 | Fibrillar Aβ stimulates TFEB translocation in a mTORC1-independent pathway. (**a**) Quantification of the proportion of cells with nuclear vs. cytoplasmic TFEB in different time points and the fluorescence signal of each cell was estimated by examining more than 50 cells. One-way ANOVA with Turkey's test, $^{**}p < 0.01$; $^{***}p < 0.001$. (**b**) fAβ-stimulated TFEB nuclear translocation in microglia does not dependent on changes of mTORC1 activity. BV2 cells were incubated with fAβ (500nM) or mTORC1 inhibitor torin1 at 37℃ and the cells were harvested and lysed at different time points, followed by Western blotting analysis with antibodies for specific phosphorylated sites of mTORC1 substrates, Ser 473 of AKT or Thr 37/Thr 46 of 4EBP1.

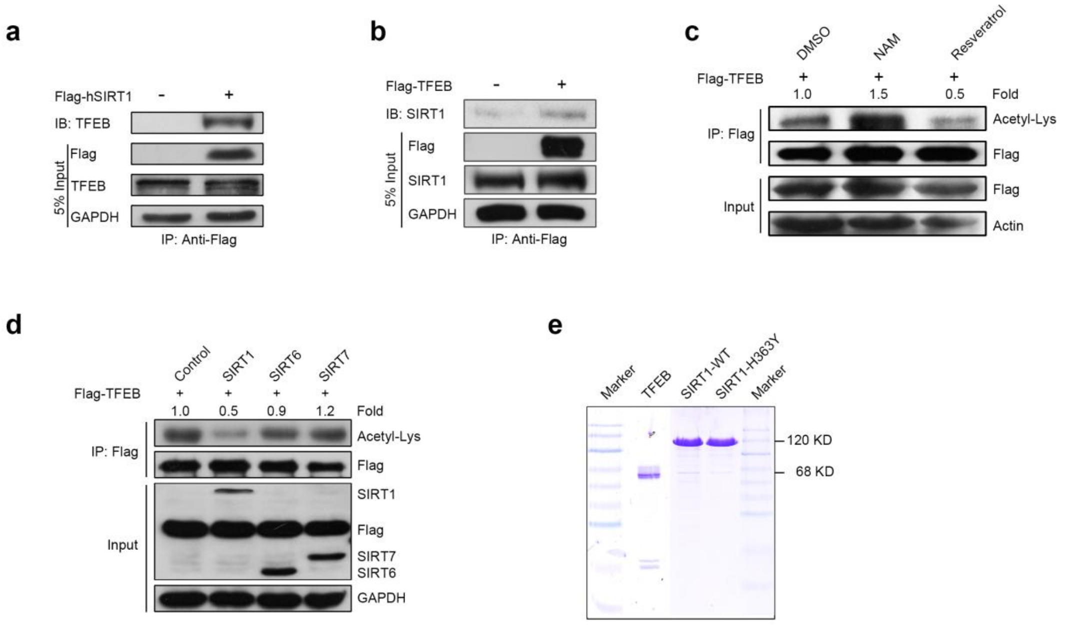

Supplementary Figure 3 | TFEB is a deacetylation substrate of SIRT1. (**a,b**) TFEB interacts with SIRT1. Coimmunoprecipitation of Flag-tagged TFEB or SIRT1 transfected into HEK293T cells for 24 h and endogenous SIRT1 or TFEB was detected by their specific antibodies, respectively. (**c**) Chemical inhibition or activation of SIRT1 increases or decreases TFEB acetylation. Flag-tagged TFEB was transfected into HEK293T cells treated with or without NAM (10mM) or Resveratrol (50μM). Acetylation levels of TFEB was measured by IP Western blotting analysis. Values were expressed as fold changes relative to TFEB-WT with DMSO treatment and normalized to IP-Flag. (**d**) SIRT1, but not SIRT6 or SIRT7, deacetylates TFEB in the nucleus. Flag-tagged TFEB was co-transfected into HEK293T cells with SIRT1, SIRT6 or SIRT7 for 24 h, respectively. Acetylation levels of TFEB was measured by IP Western blotting analysis. Values were expressed as fold changes relative to TFEB-WT with DMSO treatment and normalized to IP-Flag. (**e**) Purified TFEB, SIRT1-WT or SIRT1-HY for *In vitro* deacetylation assay. Flagged-tagged TFEB (68KD), SIRT1-WT or

SIRT1-HY (120KD) was transfected into HEK293T cells for 24 h and was immunoprecipitated with an anti-flag antibody followed by Commassie Blue R250 staining.

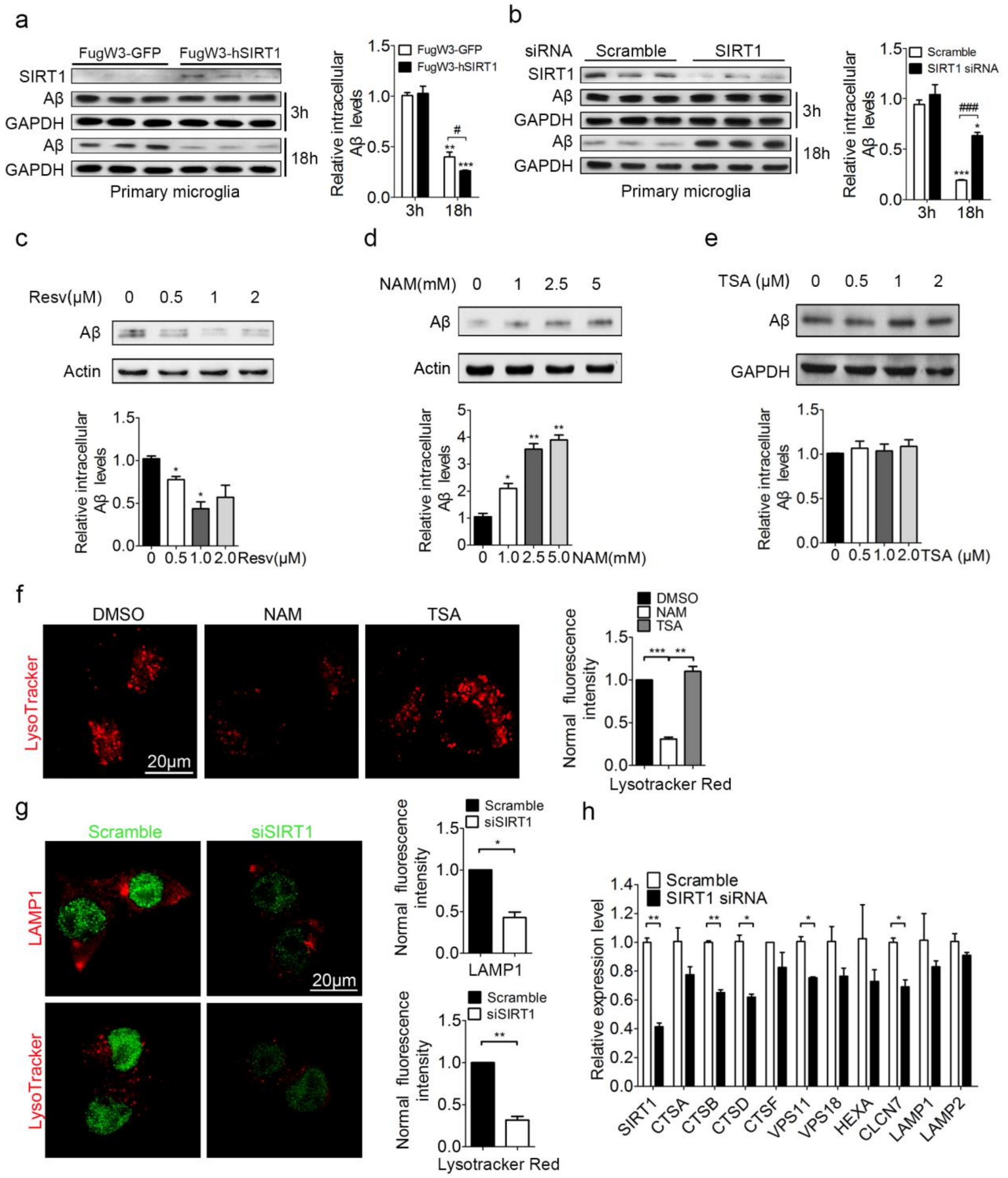

Supplementary Figure 4 | SIRT1 accelerates fAβ degradation in microglia in a TFEB-dependent manner. (**a,b**) SIRT1 accelerates fAβ degradation in primary microglia. GFP or human SIRT1 was overexpressed in BV2 cells by lentiviral system (**a**). Scramble or SIRT1 siRNA was delivered into BV2 cells for 72 h (**b**). The cells were incubated with fAβ (500nM) for 3 h or 18 h and were harvested for detection of intracellular Aβ levels by Western blotting analysis. The band intensity was measured in three independent experiments

indicating relative intracellular Aβ levels and the mean ± SEM. are shown in the right panel. Two-way ANOVA, comparison between different time points, *p< 0.05; **p< 0.01; ***p< 0.001. Unpaired Student's t-test, comparison against the Fugw3-GFP or the Scramble, #p< 0.05, ###p< 0.001. (**c-e**) Chemical activation or inhibition of SIRT1 by Resveratrol (**c**) or NAM (**d**) increases or decreases fAβ degradation in a dose-dependent manner, respectively, while TSA has no effects on fAβ degradation (**e**). BV2 cells were pretreated with Resveratrol or NAM or TSA at different concentration. The cells were then incubated with fAβ (500nM) in the presence of these drugs for an additional 18 h as in (**a,b**). One-way ANOVA with Turkey's test, *p< 0.05; **p< 0.01. (**f**) NAM, but not TSA, reduces lysosomal biogenesis in microglia. BV2 cells were pretreated with NAM or TSA, followed by staining with Lysotracker Red (Red), and the fluorescence signal of each cell was estimated by examining more than 50 cells. One-way ANOVA with Turkey's test, **p< 0.01; ***p< 0.001. (**g**) SIRT1 knockdown inhibits lysosomal biogenesis in microglia. BV2 cells are transfected with scramble or SIRT1 siRNA and stained as in (**f**) and the fluorescence signal of each cell was estimated by examining more than 50 cells. One-way ANOVA with Turkey's test, *p< 0.05; **p< 0.01. (**h**) SIRT1 knockdown downregulates TFEB induction of its target genes. Quantitative PCR (qPCR) analysis of TFEB target genes in BV2 cells scramble or SIRT1 siRNA. Values represent means ± SEM of three independent experiments. Unpaired Student's t-test, comparison against the scramble, *p< 0.05; **p< 0.01.

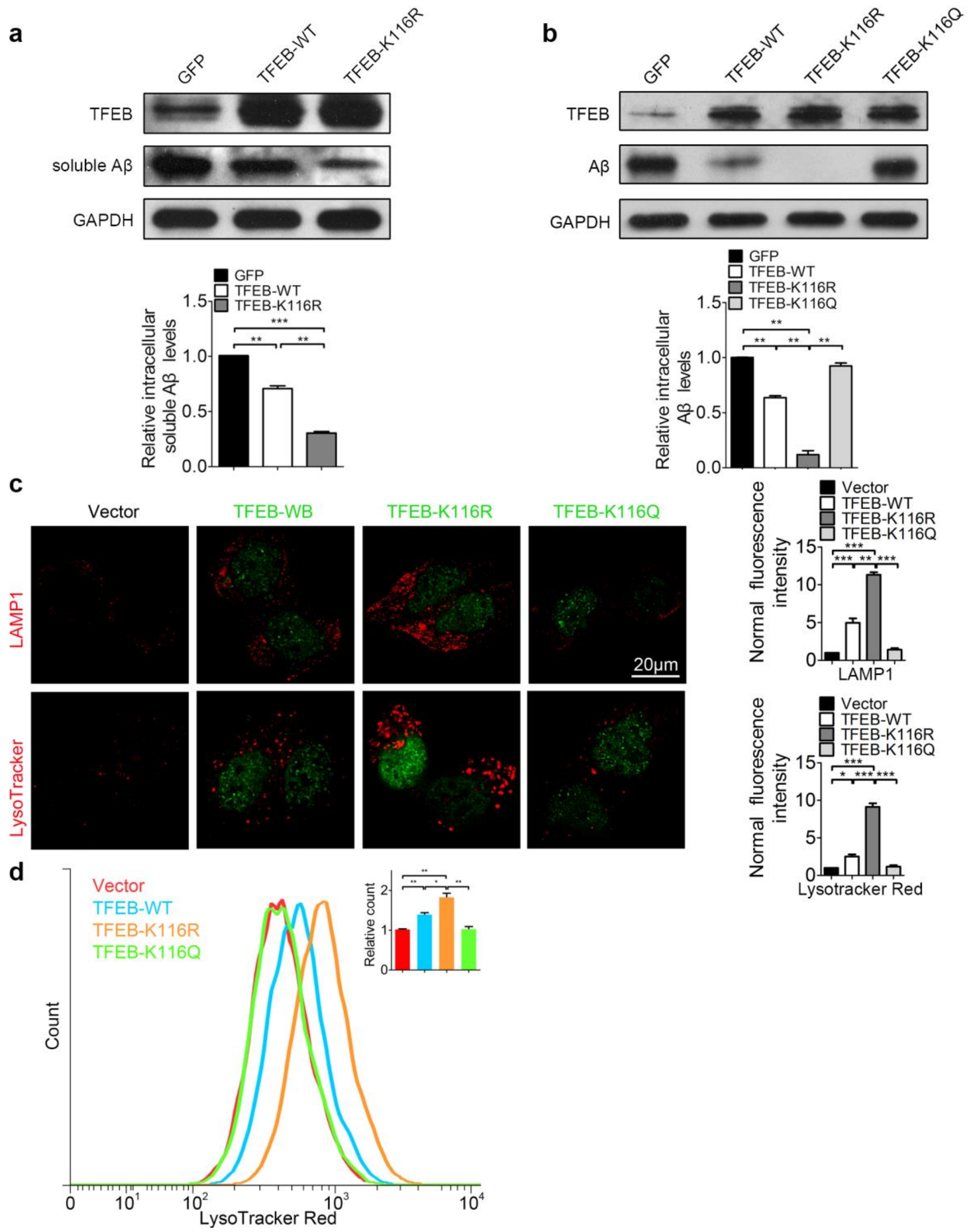

Supplementary Figure 5 | Deacetylated TFEB facilitates fAβ degradation by enhancing lysosomal biogenesis. (**a**) Deacetylation of TFEB at K116 futher facilitates sAβ degradation. BV2 cells were infected with lentivirally overexpressed FugW3-GFP or FugW3-TFEB (WT or K116R). The cells were incubated with fAβ (500nM) for an additional 18 h and were harvested for detection of intracellular Aβ levels by Western blotting analysis. The band intensity was measured in three independent experiments indicating relative intracellular Aβ

levels and the mean ± SEM. are shown in the right panel. One-way ANOVA with Turkey's test, **p< 0.01; ***p< 0.001. (**b**) TFEB-K116Q (acetylation mimic) mutant cannot facilitate fAβ degradation in microglia. The experiment was conducted and the result was analysed as in (**a**). (**c**) TFEB-K116Q mutant cannot stimulate lysosomal biogenesis in microglia. Empty vector or GFP-tagged TFEB (WT or K116R or K116Q) was overexpressed in BV2 cells, followed by staining with an antibody against LAMP1 (Red) or LysoTracker Red, respectively. The fluorescence signal of each cell was estimated by examining more than 50 cells. One-way ANOVA with Turkey's test, *p< 0.05; **p< 0.01; ***p< 0.001. (**d**) Flow cytometric analysis of lyososomes stained with LysoTracker Red in BV2 cells treated as in (**c**). Values of mean fluorescence were expressed as fold changes. One-way ANOVA with Turkey's test, *p< 0.05; **p< 0.01; ***p< 0.001.

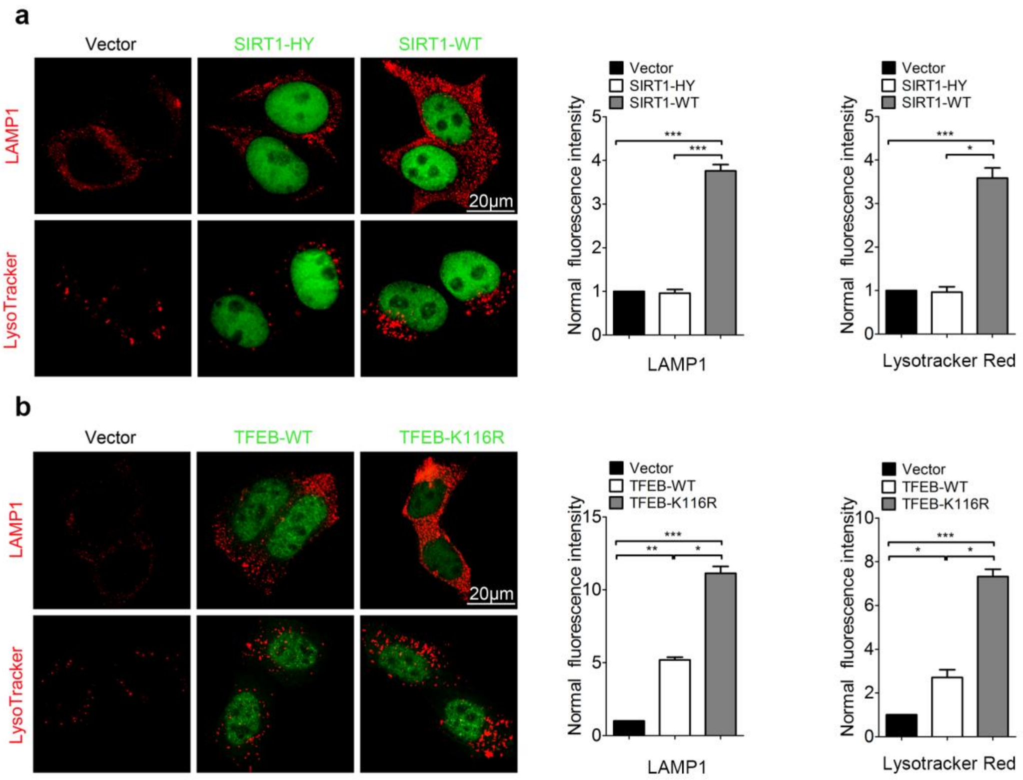

Supplementary Figure 6 | SIRT1-mediated deacetylation of TFEB facilitates fAβ degradation by enhancing lysosomal biogenesis in Hela. (**a**) SIRT1 stimulates lysosomal biogenesis in Hela. Empty vector or GFP-tagged SIRT1 (WT or HY) was overexpressed in Hela cells followed by staining with an antibody against LAMP1 (Red) or LysoTracker Red, respectively. (**b**) Deacetylation of TFEB at K116 stimulates lysosomal biogenesis in Hela. Empty vector or GFP-tagged TFEB (WT or K116R) was overexpressed in Hela cells followed by staining with an antibody against LAMP1 (Red) or LysoTracker Red, respectively. The fluorescence signal of each cell was estimated by examining more than 50 cells. One-way ANOVA with Turkey's test, *p< 0.05; **p< 0.01; ***p< 0.001.

Table S1 ChIP-qPCR primers of CLCN7

ChIP primers	Forward primer	Reverse primer
Primer1	cctggcgccatgactcatg	tcacgccattctcctgcct
Primer2	tgaggcaggagaatggcgt	tccgcctcctgggttcaaa
Primer3	ttgaacccaggaggcgga	gcagccttgacctcttggg
Primer4	aacccaagaggtcaaggctg	gtttcactgtgttagccaggatg
Primer5	tcctggctaacacagtgaaacc	tgtgcctcaccctcttcctt
Primer6	aaaggaagaggatgaggcacaa	cagctcagtgcagccttga
Primer7	gtcaaggctgcactgagct	tagccaaatagccgcccg
Primer8	ggcggctatttggctaattcac	gtcccgaagcatgcatcc
Primer9	cggatgcatgcttcggga	gagtcttgggaggcgaatgg
